# Diagnostic challenges of neurosarcoidosis in non-endemic areas

**DOI:** 10.3389/fneur.2023.1220635

**Published:** 2024-01-11

**Authors:** Keshari Shrestha, B. K. Kleinschmidt-DeMasters, D. Ryan Ormond

**Affiliations:** ^1^Department of Neurosurgery, University of Colorado School of Medicine, Aurora, CO, United States; ^2^Department of Pathology, University of Colorado School of Medicine, Aurora, CO, United States

**Keywords:** autoimmune disease, neurosarcoidosis, sarcoid, unusual presentation, coexistent meningioma

## Abstract

**Background:**

Neurosarcoidosis (NS) is a challenging diagnosis, particularly when cases occur in low-prevalence, non-endemic geographic regions. In the United States, the highest incidence is in the Midwest and Northeast, compared to our Southwest location. While it is well known that NS may clinically and neuroradiographically mimic meningeal carcinomatosis, autoimmune or infectious pachymeningitis, neurosyphilis, or tuberculosis, diagnosis may be particularly challenging if systemic signs of sarcoidosis are lacking or unconfirmed or if dural-based masses are present. We reviewed our Colorado experience with NS cases, focusing our study on cases where NS represented the first histological confirmation of disease.

**Methods:**

A search of departmental databases was conducted with the search term “neurosarcoidosis” to identify cases 1-2008 to 12-2019, inclusive of the given case numbers. Patients were only included if their clinical and neuroimaging features were unusual and only when a biopsy of the central nervous system (CNS) represented the first confirmed diagnosis of sarcoidosis.

**Results:**

A total of 17 cases were identified, of which the biopsy of the CNS was used for the initial confirmation of the disease in 9 of them. The most unusual findings were two patients with dural-based masses, one of which had pure NS as the cause of meningioma-like lesions and the second of which had coexistent meningioma and intimately admixed non-necrotizing granulomas of NS.

**Conclusion:**

NS with unusual features, especially in non-endemic areas, continues to yield diagnostic challenges for neurologists, neuroradiologists, and pathologists.

## Introduction

Sarcoidosis is a multi-organ inflammatory disorder of unknown etiology characterized by the formation of small, tight, compact, non-caseating granulomas often containing multinucleated giant cells (1). This disease shows a striking geographical predilection, with the highest prevalence in the United States (US) in the Midwest and Northeast areas (2). An ethnic predisposition in those of black or African American ancestry has been recognized for several decades, with a threefold greater prevalence cited in black people (3). Interestingly, a very recent brief epidemiology report suggested that “geographical racial variation in the United States may explain the spatial clustering of sarcoidosis prevalence across the United States (2).” However, the same study noted “that significant clusters were identified (regardless of race) near metropolitan areas (2).” Thus, the effect of environmental and other factors still requires further analysis (2), as does possible race/ethnic predilection in urban areas.

Sarcoidosis, especially isolated organ involvement types such as neurosarcoidosis (NS), is far less likely to be high on the list of differential diagnoses for patients in the non-endemic areas of the US, such as our Southwestern US region, than for those in the areas of high prevalence. While sarcoidosis most commonly involves the lungs, skin, and eyes (4), sarcoid can affect any organ system in the body (5, 6), and is highly diverse when involving the nervous system (7–9). NS is identified in 5–10% of patients, in half of which it may be the initial clinical presentation (10).

NS may involve brain parenchyma, cranial nerves, meninges, spinal cord, and/or peripheral nerves, with cranial nerve involvement being the most prevalent, occurring in 50–70% of cases (11–13). Common or “usual” neurologic manifestations include cranial nerve palsy, peripheral neuropathy, seizures, and neuropsychological and cerebellar symptoms. Less common clinical symptoms include hydrocephalus and aseptic meningitis (6). Typical imaging findings of NS include multiple white matter lesions coupled with meningeal enhancement or “sugarcoating” near the skull base, as well as focal nodular enhancement (14).

Given our low prevalence in a Southwestern US location [Colorado, with approximately a twofold relative risk decrease over the southeast and northeast regions of the country (2)], albeit in an urban setting (Denver), we reviewed our 17 years experience with biopsy-proven NS, highlighting its clinical and neuroimaging features, particularly in cases for which the nervous system biopsy was the first tissue documentation of sarcoidosis. We additionally queried our database to see if our experience in a non-endemic region of the country suggested a strong African American prevalence that might have assisted, in retrospect, in making the diagnosis of NS. Conversely, we were interested in seeing if our cases from the greater metropolitan Denver area had existed, “regardless of race, near metropolitan areas (2).” Unfortunately, our small case numbers did not allow for a statistical evaluation of these possibilities. A review of neuroimaging features highlighted cases in which intracranial mass lesions, particularly of the dura, might have prompted further diagnostic confusion.

## Materials and methods

### Patient population

The requirement of ethical approval was waived by the Colorado Multiple Institutional Review Board for this study because it was determined that it met the criteria for exemption under Category 4. The studies were conducted in accordance with local legislation and institutional requirements. The ethics committee/institutional review board also waived the requirement of written informed consent for participation from the participants or the participants’ legal guardians/next of kin because it qualified for exemption under Category 4. Under this institutional review board-approved protocol (COMIRB 21–2,529), databases of the Departments of Neurosurgery and Pathology, University of Colorado Health Science Center, Aurora, CO, were searched by the search term “neurosarcoidosis” to identify cases 1-2008 to 12-2019, inclusive of the given case numbers. After further review of our total cases (*n* = 17), a subset in which the nervous system biopsy had provided the first proven diagnosis of sarcoidosis was further studied in detail (*n* = 9). All nine cases had received care at either the University of Colorado Hospital or Children’s Hospital Colorado. Medical records were assessed for patient demographics, ethnicity (as self-declared), treatment, and outcome, with data summarized in the table format.

## Results: Clinical and neuroimaging features

The total cohort consisted of 17 patients, 6 male individuals, and 11 female individuals, with declared ethnicity; of the 17 patients, 7 were Caucasian, 5 were African American, 4 were Hispanic, and 1 was of Hawaiian-Pacific Islander ethnicity. The cohort on which we focused further consisted of 9 patients, 4 male individuals, and 5 female individuals, with declared ethnicity; of the 9 patients, 3 were Caucasian, 3 were African American, and 3 were of Hispanic ethnicity. These patients ranged in age from 13 to 66 years. Imaging typically demonstrated these lesions well with an inflammatory infiltrate opening the blood–brain barrier, creating edematous lesions with contrast enhancement. The findings are summarized in the table, but brief clinical histories are also detailed below to highlight the diverse clinical presentations.

## Case 1

A 40–45-year-old woman with no significant past medical history presented to the emergency department with 2 months of intermittent right forehead numbness. Several weeks prior, she had been treated for suspected Bell’s palsy with valacyclovir and steroids without improvement. Laboratory studies, including a serum angiotensin-converting enzyme (ACE) level, erythrocyte sedimentation rate (ESR), serum calcium, and endocrinological studies were normal. Imaging studies were suspicious for a right-sided meningioma, showing an extra-axial dural-based enhancing mass with invasion into the sella, causing possible involvement of the pituitary gland extending into the cavernous sinus. Following a right-sided dural-based mass biopsy, it was revealed from the evaluation that the patient’s granulomas were small, tight, and non-necrotizing, which was consistent with NS.

## Case 2

**Figure 1:**
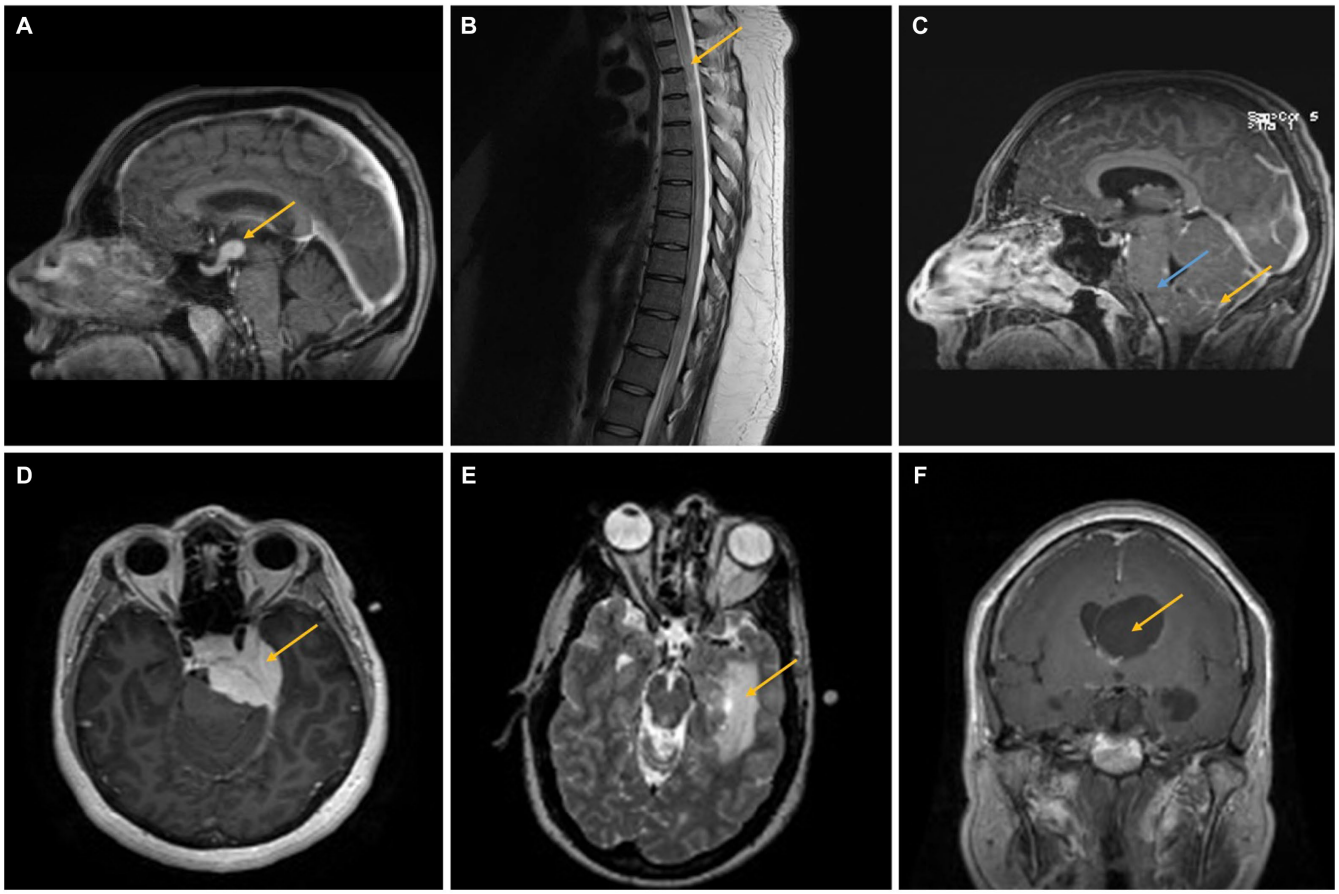
**(A)** A 40–45-year-old woman presented with headaches, nausea, vomiting, and altered mental status. Imaging showed a 1.5 cm infundibular/hypothalamic mass, which was confirmed as NS after biopsy (case 2, yellow arrow). **(B,C)** A 25–30-year-old man presented with headaches and weakness/sensory changes in the left upper and lower extremities. Imaging showed a T2 hyperintense lesion extending from T1–T12 (**B**, yellow arrow) as well as leptomeningeal enhancement (yellow arrow) and brainstem swelling (blue arrow), confirmed as NS after biopsy **(C)** (case 3). **(D)** A 35–40-year-old woman presented with headaches, facial pain, and weakness/numbness in the bilateral upper and lower extremities. MRI findings demonstrated a large, homogenously enhancing lesion along the medial aspect of the middle fossa causing compression of the left temporal lobe and pons, confirmed as a meningioma with intimately admixed small, compact granulomas consistent with NS in the resection specimen. The two comingled processes cannot be distinguished on neuroimaging (case 4, yellow arrow). **(E)** A 35–40-year-old man presented with a left medial temporal mass concerning a glioma. Biopsy instead demonstrated NS (case 5, yellow arrow). **(F)** A 55–60-year-old woman presented with right eye optic neuropathy, right temporal visual field loss, cough, and transient neurologic symptoms. Imaging showed asymmetric enlargement of the lateral ventricles with a suspected foramen of Monro obstruction (yellow arrow). The biopsy was consistent with NS (case 6).

A 40–45-year-old woman with no significant past medical history presented with headaches, nausea, vomiting, and altered mental status. On examination, it was found that she had no focal neurologic deficits. An additional investigation confirmed panhypopituitarism, and imaging results showed a substantial intra-axial mass centered in the hypothalamus that was increased with contrast and had a significant amount of surrounding edema that extended 1.5 cm into the infundibulum (Figure 1A). There was no evidence of intrathoracic sarcoidosis on chest imaging. The results of laboratory tests of infectious/autoimmune clinical markers, including serum ACE, ESR, and serum calcium, were normal. After 4 days of hospitalization, the patient underwent a right frontal endoscopic biopsy of the hypothalamic lesion and a procedure for the placement of a right frontal external ventricular drain (EVD). Pathology was consistent with NS. After starting on a tapered high-dose course of hydrocortisone, the patient was switched to 7.5 mg of prednisone per day. From a neurology standpoint, the patient’s condition improved after starting therapy.

## Case 3

A 25–30-year-old man with a history of autistic spectrum disorder complained of headaches, weakness, or sensory changes in his left upper and lower extremities, suspected to be due to transverse myelitis rather than neuromyelitis optica following an upper respiratory tract infection. After 3 months, he presented with urinary retention, weakness, sensory changes in his left upper and lower extremities, and gait instability. A T2-hyperintense intra-axial expansile spinal cord lesion ranging from T1 to T12 was seen on magnetic resonance imaging (MRI) (Figure 1B). The patient was started on 1 g of solumedrol intravenously (IV) for 5 days, followed by an oral steroid taper. After 3 months, the patient displayed increased gait instability, bilateral headaches, and tremors in both his upper and lower extremities. Due to spasticity and ataxia at this point, the patient required bilateral lower extremity support. The results of extensive laboratory studies, including an infectious and autoimmune workup, including serum ACE, ESR, and calcium, were normal. Cerebrospinal fluid (CSF) analysis revealed an increased protein concentration and the presence of oligoclonal bands. Following CSF analysis, MRI showed abnormal leptomeningeal enhancement, brainstem enlargement, compression of the fourth ventricle with modest dilation of the lateral and third ventricles, and T2 hyperintense lesions affecting the left dorsal midbrain and periaqueductal gray matter (Figure 1C). Ultimately, a biopsy was performed on the patient, which confirmed NS. The patient received infliximab infusions every 4 weeks for 7 months. After that, his mobility and neurologic problems significantly improved, and he switched to receiving infliximab infusions every 6 weeks.

## Case 4

A 35–40-year-old woman presented with intermittent symptoms including headache, facial pain, and weakness/numbness of bilateral upper and lower extremities. MRI demonstrated a large, homogeneously enhanced extra-axial mass along the medial aspect of the middle cranial fossa, causing compression of the left temporal lobe and pons without extension into the left internal auditory canal (IAC) (Figure 1D). Repeated imaging 2 months later revealed enlargement of the tumor lesions, extending into the left IAC and cavernous sinus, and possible pituitary involvement. During this period, a chest computed tomography (CT) revealed evidence of a significant right axillary lymphadenopathy related to sarcoidosis, lymphoproliferative disease, or infection. The results of laboratory tests were normal, including workups for infectious and autoimmune diseases (including ACE, ESR, and calcium). The patient then underwent a craniotomy for tumor debulking, which following the procedure showed a WHO Grade 1 meningioma intimately admixed with small, compact, non-necrotizing granulomas containing multinucleated giant cells, consistent with NS. This suggests that meningioma and NS were diagnosed at the same time. Following initial sarcoidosis treatment and after achieving lesion and symptom stability, two more procedures were needed over the course of the following 2 years.

## Case 5

A 35–40-year-old man with no significant prior medical history revealed a partially enhancing mass lesion on the left medial temporal side, which was likely a glioma. The uncus, hippocampus, basal ganglia, and choroid plexus were among the regions of the left temporal lobe affected by T2 hyperintensity, which was shown to be expansive in nature (Figure 1E). CT chest/abdomen/pelvis showed a calcified granuloma in the right lower lobe of the lung and bilateral hilar adenopathy, indicative of a granulomatous process. A few months later, the patient had intermittent vertigo, stomach pain, and numbness on the right side of his body. An infectious and autoimmune workup (including ACE, ESR, serum calcium, etc.) was normal. Later, an open biopsy revealed multifocal, noncaseating granulomas with tiny yeast forms that were suggestive but not conclusive. Histoplasmosis was treated empirically until NS was identified after a fungus-specific PCR test yielded a negative result and he failed to demonstrate any clinical improvement. The patient experienced mild postoperative right-sided weakness and memory deficits, but these symptoms gradually returned to normal with sarcoidosis therapy.

## Case 6

**Figure 2:**
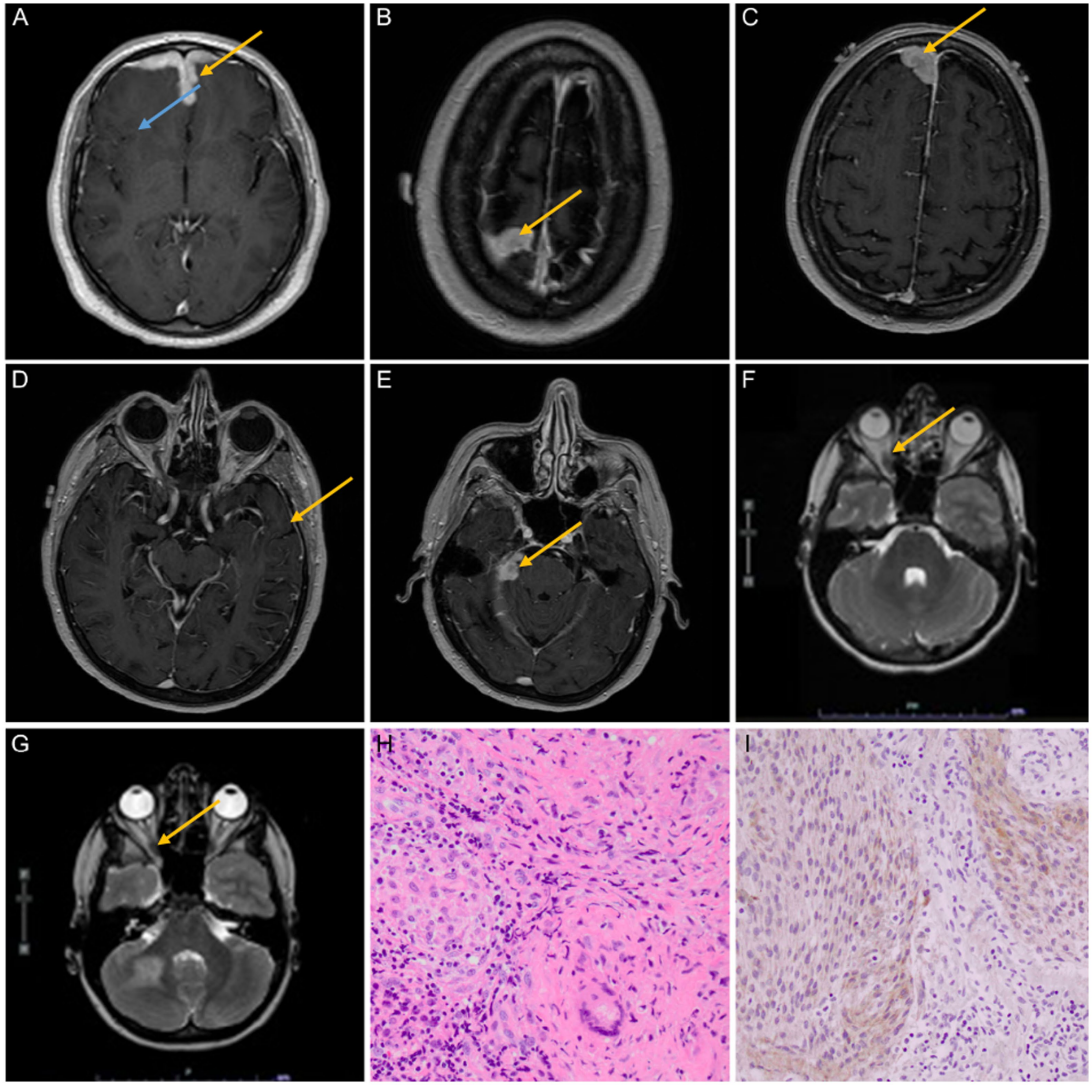
**(A–E)** A 40–45-year-old man presented with fatigue, altered mental status, short-term memory loss, right eye pain, and hemoptysis. MRI findings at multiple levels showed bilateral frontal lobe edema (blue arrow) and leptomeningeal enhancement (yellow arrows); the biopsy was consistent with NS (case 7). **(F,G)** A 65–70-year-old woman presented with a 1 cm neck mass and monocular vision loss in the left eye. MRI findings revealed three intracranial homogeneously enhancing dural masses that were believed to be meningiomas prior to biopsies. The first biopsy of the convexity lesion demonstrated dural NS; the optic nerve sheath biopsy (yellow arrows) showed only fibrosis but no granulomas or meningioma; and the third lesion has been followed expectantly as a possible meningioma but was unbiopsied (case 8). **(H,I)** The resection specimen on patient 4 showed an intimate admixture of small, compact, non-necrotizing granulomas containing typical multinucleated giant cells of sarcoidosis (at right, **H**) and meningioma, WHO grade 1 (at left, **H**) (hematoxylin and eosin, 400×). Meningioma was further verified by immunoreactivity for epithelial membrane antigens (**I**, 400×).

A 55–60-year-old woman presented with right eye optic neuropathy, right temporal visual field loss, cough, and a few other transient symptoms such as right-sided hearing loss, vestibular dysfunction, and weakness/numbness in her extremities. A chest CT showed multiple pulmonary nodules, consistent with a granulomatous process. A clinical diagnosis of possible pulmonary sarcoidosis and NS was made based on MRI findings of enhanced coating and enlarging CN VII and VIII (Figure 2A). The results of laboratory studies, including ACE, ESR, serum calcium, and other inflammatory/autoimmune markers, were normal. However, in order to stabilize her hearing impairment and treat her vestibular dysfunction, a 7.5 mg maintenance dose of prednisone taper was initiated in light of the imaging results. Several years later, the patient presented with obstructive hydrocephalus with an enhancing cystic lesion obstructing the ventricle at the foramen of Monro and underwent endoscopic cyst fenestration, septostomy, and biopsy, with pathologic findings consistent with NS. Following the initiation of a daily dosage of 5 mg of prednisone and a weekly dosage of 15 mg of methotrexate, the patient’s clinical symptoms resolved and her imaging results improved.

## Case 7

A 40–45-year-old man presented with new-onset fatigue, altered mental status, short-term memory loss, right eye pain, and hemoptysis for 4 days, along with night sweats for months and a 20-pound weight loss. The MRI report showed bilateral frontal lobe edema (expansile T2 hyperintense disease) and diffuse dural thickening with leptomeningeal enhancement (Figures 2B–E). CT chest/abdomen/pelvis demonstrated calcified mediastinal and hilar lymph nodes indicative of a prior granulomatous process. The results of an infectious and autoimmune workup, including ACE, ESR, and serum calcium, were normal. Ultimately, an open biopsy was consistent with NS. After receiving treatment for seizures and sarcoidosis, the patient’s symptoms improved.

## Case 8

A 65–70-year-old woman presented with a 1 cm neck tumor and monocular vision loss in her left eye (retrospective slides were collected and reviewed at our institution, revealing a granulomatous lesion). On MRI, there were three intracranial homogeneously enhancing dural-based extra-axial masses (one mass to the right of the superior sagittal sinus, one mass on the midline involving convexity dura, and one mass involving the tentorium cerebelli on the right side) that were judged to be most consistent with meningiomas based on neuroimaging. The patient was found to have an increasing extra-axial tumor involving the left optic nerve sheath, which raises the possibility of meningioma (Figures 2F,G). Significant mediastinal and hilar lymphadenopathy was seen on the CT chest. The results of infectious and autoimmune workups were insignificant. After the rapid growth of her tumors a few years later, she underwent a craniotomy, and in the convexity lesion, there was granulomatous dural inflammation consistent with NS. A small biopsy of the left optic nerve sheath mass showed only fibrosis but no granulomas or meningioma; this was likely to be NS. A biopsy was not performed on the third lesion in the sagittal sinus. In the end, sarcoidosis treatment resulted in symptom stability.

## Case 9

A 10–15-year-old young patient with a history of an upper respiratory tract infection 1 week prior presented to the emergency department of our hospital after an episode of unilateral facial weakness/droop and aphasia that lasted 30 min. He showed no neurological damage upon physical examination. An MRI demonstrated T2 hyperintense, expansile periventricular lesions with wispy enhancement concerning a demyelinating process, initially diagnosed as relapsing and remitting multiple sclerosis (Figure 2H, imaging unavailable). Laboratory studies for infectious and autoimmune diseases, including ACE, ESR, and serum calcium, were normal. The results of the biopsy revealed neurosarcoidosis, which was stabilized clinically and on imaging after treatment.

## Results: Histological findings

All patients’ biopsies demonstrated multiple small, tight, compact, non-necrotizing granulomas with multinucleated giant cells, consistent with NS. Most biopsies had been further assessed using stains for fungi (Grocott’s methenamine silver stain) and periodic acid-Schiff (PAS), with select cases further stained with Fite’s Stain Kit for acid-fast bacilli, Gram Stains kit for bacteria, and Verhoeff-van Gieson (VVG) Stain for elastic fibers, to exclude granulomatosis with polyangiitis (formerly Wegener granulomatosis). Two cases (patients 5 and 8) had further confirmation of the absence of an infectious etiology by negative polymerase chain reaction testing.

The most unusual cases, according to histological reports, were patients 4 and 8. The first had a dural-based mass that was resected and proved to be a meningioma, meningothelial type, World Health Organization (WHO) grade 1 benign, with intimate admixture of numerous small, compact, noncaseating granulomas within the meningioma (Figure 2H). The meningioma was further proven by immunoreactivity for epithelial membrane antigen (Figure 2I) and somatostatin receptor 2A (data not shown). Given the complete admixture of the two processes, unsurprisingly, the two components could not be distinguished on preoperative neuroimaging (see Figure 1D). Patient 8 had three intracranial enhancing dural-based masses (one mass to the right of the superior sagittal sinus, one mass on the midline involving convexity dura, and one mass involving the tentorium on the right side) concluded from pre-biopsy neuroimaging to be meningiomas and an enhancing left optic nerve sheath mass. The right convexity mass was resected and proved to be NS without admixed or adjacent meningioma; a subsequent biopsy of the left optic nerve sheath mass was non-diagnostic. While the latter showed fibrosis and no granulomas, it also did not contain meningioma. The third mass has been followed expectantly without resection but may represent a true meningioma. A biopsy of a right submandibular lymph node on this patient at another institution was obtained retrospectively after these two biopsies of the intracranial lesions at our institution were found to contain non-necrotizing granulomas and, in retrospect, to be affected by sarcoidosis also.

## Results: Summary of clinical outcomes

The majority of our patients (66.7%) were treated initially with either intravenous or oral steroids. Three patients (33.3%) were treated with infliximab for stabilization or resolution of their symptoms. One patient was initially treated with infliximab but was switched to azathioprine due to its lack of efficacy. Two patients (22.2%) were treated with methotrexate with improvement in their symptoms, and one patient who was initially treated with methotrexate was switched to hydroxychloroquine and infliximab. One patient was adequately treated with azathioprine alone (11.1%), and one patient was briefly treated with rituximab (11.1%) without adequate symptom improvement. The majority of patients (55.6%) were treated with multiple agents. Eight out of nine patients (88.9%) experienced clinical improvement or stabilization with therapy, and one patient was lost to follow-up with treatment elsewhere.

## Discussion

We detailed nine care reports of patients in whom a CNS biopsy was the first documentation of sarcoidosis. This study represented 9 of 17 NS patients encountered at our institution during the study period, roughly in half of all cases. The number of cases in which NS was the first documented presentation of sarcoidosis parallels findings in the literature (10). In our nine patients, initial imaging findings often mimicked other disease processes similar to those described elsewhere (1, 9, 15, 16). After chart review, it became apparent that alternate etiologies for the diverse presentations might be favored by a clinician when occurring in an area of the United States where NS is uncommon, such as our own. Thus, the diagnosis of NS in non-endemic areas may be especially challenging.

Extensive testing to exclude alternate etiologies was usually required in our patients, as it usually is in patients with NS. Ancillary chest imaging, pulmonary function tests, serum chemistries (angiotensin-converting enzyme, calcium, soluble interleukin-2 receptor), and cerebrospinal fluid (CSF) polymerase chain reaction (PCR) and cytology may be required to rule out other etiologies (9, 17). A definitive diagnosis requires biopsy evidence of non-caseating granulomas and the absence of an infectious etiology (18).

The clinical presentations of most, but not all, of our nine patients were generally consistent with a previous study describing 1,088 patients with NS diagnosed between 1965 and 2015, in which 55% of the patients presented with cranial nerve involvement and 33% presented with headache (15). Less common presenting symptoms highlighted within this case series include neuroendocrine involvement, hydrocephalus, and psychiatric symptoms, all with a reported prevalence of less than 10% (15). In our case series, cases 4 and 9 demonstrated vascular involvement in the form of a cerebral vascular accident and dural venous thrombosis, respectively. Vascular involvement has an estimated prevalence of 6% (17), with dural venous thrombosis reported as an extremely rare manifestation (16). Additionally, in case 9, we presented the report of a patient who experienced reversible aphasia, a rare clinical presentation.

While none of the nine patients on whom we focused our study had documented sarcoidosis in other organ sites, several had findings that, after sarcoidosis was proven, likely confirmed the disease in retrospect. One patient (patient 6) initially presented with simultaneous neurologic and pulmonary symptoms of sarcoidosis, which led to a CT workup showing evidence of pulmonary nodules. While there was a high suspicion of NS in this patient, a definitive diagnosis was made only after a neurosurgical tissue biopsy. Four out of nine patients (44.4%) had imaging evidence of pulmonary sarcoidosis. Two out of nine patients (22.2%) had extra-pulmonary lymphadenopathy, suggesting a possible inflammatory/granulomatous process. In patients with concomitant pulmonary or lymphatic involvement, neurosarcoidosis was high on the differential diagnosis. Out of our nine patients, 66.7% had undergone a pre-biopsy negative infectious disease workup, 55.6% underwent a negative autoimmune workup, and 44.4% underwent CSF serologies in order to rule out other causes of disease. All nine patients required a tissue biopsy for confirmation of NS.

Within this case series, the two patients with the most unusual features were patients 4 and 8. Patient 8 had three dural-based lesions that were believed to be meningiomas. Resection proved NS in one, while the left optic nerve sheath mass resulted in a non-diagnostic biopsy showing only fibrosis but no granulomas or meningioma; the third lesion was not resected. Dural-based, nodular NS-mimicking meningiomas are rare, but multiple case reports exist in the literature (19–23). However, the finding in patient 4 of intimately admixed non-necrotizing granulomas of NS and meningothelial meningioma is unique to our knowledge, although other autoinflammatory or autoimmune diseases coexisting with tumors have been described elsewhere (24–26). This novel finding raises several considerations. Namely, the question arises as to whether there was a tropism of NS to the meningioma or if this relationship is purely coincidental. We favor the former since at least one example of sarcoidosis within a meningioma has been previously published (27). Of note, meningiomas, and pituitary adenomas are the two most frequent types of tumors that serve as recipients of tumor-to-tumor metastases, presumably due to their benign nature and rich vascularity (28). Thus, the notion that the meningioma might also be a “recipient” of NS due to the rich vascularity of the meningioma has some precedence.

In terms of ethnicity, among all 17 of our NS cases, 7 were Caucasian, 5 were African American, 4 were Hispanic, and 1 was a Hawaiian-Pacific Islander. The cohort on which we focused further consisted of 9 patients, with declared ethnicity of which 3 were Caucasian, 3 were African American, and 3 were of Hispanic ethnicity. Although we did have a larger number of persons of African American ethnicity in our entire NS patient population than might have been predicted based on the ethnic distribution for our Denver metropolitan area (64.6% Caucasian, 5.29% African American, 22.6% Hispanic, https://www.metrodenver.org/regional-data/demographics/ethnicity), it was not sufficiently skewed to aid us in diagnosing NS in our non-endemic region.

In terms of outcome, the majority of our patients (66.7%) were treated initially with either intravenous or oral steroids. Three patients (33.3%) were treated with infliximab for stabilization or resolution of their symptoms. One patient was initially treated with infliximab but was switched to azathioprine due to its lack of efficacy. Two patients (22.2%) were treated with methotrexate with an improvement in their symptoms, and one patient who was initially treated with methotrexate was switched to hydroxychloroquine and infliximab. One patient was adequately treated with azathioprine alone (11.1%), and one patient was briefly treated with rituximab (11.1%) without adequate symptom improvement. The majority of patients (55.6%) were treated with multiple agents. Eight out of nine patients (88.9%) experienced clinical improvement or stabilization with therapy, and one patient was lost to follow-up with treatment elsewhere. The favorable response of NS to therapies highlights the value of making an early and accurate diagnosis, even in instances where neuroimaging features are unusual, a known history of systemic sarcoidosis has not been established, and the disease occurs in geographic regions with a low known prevalence.

## Conclusion

Neurosarcoidosis is a challenging diagnosis to make and maybe even more so in non-endemic areas.

Rarely, neurosarcoidosis presents as a dural-based mass(es) mimicking meningiomas, and even rarer is the intimate admixture of NS within a meningioma.

## Ethics statement

The requirement of ethical approval was waived by the Colorado Multiple Institutional Review Board for this study because it was determined that this study met criteria for exemption under category 4. The studies were conducted in accordance with the local legislation and institutional requirements. The ethics committee/institutional review board also waived the requirement of written informed consent for participation from the participants or the participants’ legal guardians/next of kin because it qualified for exemption under category 4.

## Author contributions

KS, BK-D, and DO were involved in researching primary material and editing the manuscript. KS wrote the initial draft. DO oversaw scientific rigor and designed the study. All authors contributed to the article and approved the submitted version.
